# Social support as perceived, provided and needed by family-members of migrants with type 2 diabetes – a qualitative study

**DOI:** 10.1186/s12889-024-19101-9

**Published:** 2024-06-17

**Authors:** Jenny Stenberg, Katarina Hjelm

**Affiliations:** 1 Department of Public Health and Caring Sciences, Uppsala University, PO Box 564, Uppsala, S-751 22 Sweden; 2 Department of Medical Sciences, Uppsala University, Uppsala, Sweden

**Keywords:** Foreign-born persons, Family members, Informal caregivers, Individual perceptions, Qualitative study, Semi-structured interviews, Social support, Type 2 diabetes mellitus

## Abstract

**Background:**

Social support provided by a family member has been found to have a buffering effect on distress and is associated with better diabetes self-care. This study explores the meaning of social support, as described by close family members of foreign-born people living with type 2 diabetes (PWDM) in Sweden. It also explores the support provided by family members, and the support they need to be able to support the PWDM.

**Methods:**

Qualitative explorative study, semi-structured interviews for data collection. Qualitative content analysis based on a theoretical framework on social support. Purposive sample of 13 family members, 18–52-years-old, born in countries in the Middle East, Africa, and Russia.

**Results:**

The meaning of support was described as social and emotional. Most participants described a stressful situation; feelings of sadness/worry about the risk of the PWDM developing complications added to a strained life situation from which one could not opt out. Frequent daily contacts in a family network were evident, particularly by children trained as healthcare professionals. Caring for a family member was considered a filial piety, but it was also a chance to reciprocate. The support provided was mainly informational (e.g., reminders about nutritional intake), but it was also instrumental/practical (administering medicines, helping with economy/logistics, planning/cooking meals, basic care) and emotional (sharing meals, thoughts, and activities). The support the family members needed was getting first-hand information by attending the physician visits, being able to book appointments themselves at suitable times, and preventing the withholding of important information about the PWDM. They also desired an open telephone-line, oral and written information, particularly on diet.

**Conclusions:**

To family-members, supporting the PWDM was normal and a filial piety. Support provided and needed was not only informational but also instrumental/practical and emotional. In diabetes care, addressing foreign-born individuals, diabetes education needs to be developed, also including family members. Informational material, particularly on diet, and improved access to healthcare and information about the healthcare system have the potential to increase family members’ control over the situation and prevent a negative trajectory in caregiving with perceived demands causing high levels of stress.

## Introduction

The management of diabetes necessitates an active role by the patient. Social support provided by a family member or close friend has been found to have a buffering effect on distress. Such support is associated with greater diabetes management self-efficacy and better self-monitoring of blood glucose [1–4]. Hence, in health care, and diabetes care in particular, close relatives are a resource highly needed, however, underutilised [5]. Nonetheless, knowledge about what kind of support family members can contribute and what support they need themselves to be able to contribute positively to the person with diabetes health is limited. Little is known about the meaning of support, as perceived by family members of foreign-born people living with type 2 diabetes (PWDM). With the exception of a study exploring the types of support accessed by Asians in the UK living with diabetes or coronary heart disease, there are no other studies in this area [6]. The family was a source of emotional support and physical care, and participants identified barriers and facilitators to the maintenance of a healthy lifestyle. Self-management is shaped by networks of relationships outside the formal healthcare system; however, this has been studied to a limited extent. Hence, there is a need to gain more knowledge about what kind of support family members can contribute and what support they need to be able to contribute positively to the PWDM’s health.

## Background

### Diabetes management

Prevention of type 2 diabetes and diabetes complications, patient education, and control of blood glucose levels are core elements in diabetes care [7]. Hence, the management of diabetes necessitates an active role by the patient. This involves lifestyle modifications, such as improving diet, increasing physical activity, self-monitoring of one’s health status (blood glucose and examination of feet), acquisition of diabetes knowledge, as well as adherence to professional advice [4]. The self-management of type 2 diabetes is a cornerstone to achieving good glycaemic control and reducing the risk of developing microvascular (retinopathy, nephropathy, and neuropathy) and macrovascular (cardiovascular and cerebrovascular disease) complications.

### Diabetes mellitus type 2 – a pandemic

The proportion of people with type 2 diabetes mellitus is increasing in most countries, but the burden of diabetes, both in terms of prevalence and the number of adults affected, has increased faster in low-income and middle-income countries than in high-income countries [8]. There are an estimated 463 million adults living with diabetes worldwide. Since 1980, there has been a near quadrupling of the number of cases. By 2045, the number is expected to increase to 700 million, equalling 9.9% of the world’s population [9]. In Sweden, the prevalence of diabetes is considerably higher among immigrants from non-European countries than the general population, primarily among immigrants from the Middle East [10]. Diabetes and its complications have a significant impact on individuals and their families, health systems, and national economies; moreover, the estimated global, direct health expenditure on diabetes is expected to increase further [11].

### Migration and diabetes

Migration is increasing, both worldwide and to Sweden. The number of foreign-born people in Sweden has increased from 11% of the registered population in 2000 to over 20% in 2022 [12]. People who have immigrated to Sweden come from various backgrounds, countries, at different times and with different reasons for immigration. The foreign-born population is therefore characterised by great diversity. The group of persons born outside Europe who have spent 0–9 years in Sweden are characterised largely by the refugee immigration that took place in recent years. In this group, Syria is the most common country of birth, but many immigrants have spent a long time in Sweden; other common countries of birth are Iraq, Iran, North Africa, Finland, Poland and countries of the former Yugoslavia [13].

In Sweden, 8 out of 10 people think they are in good or very good health, and there are no differences between different groups of foreign-born and Sweden-born individuals [13]. Overall, the health of immigrants may often be better than that of the native population upon arrival to the new country. The migrating populations are also, in general, in better health than those of the same populations remaining in the country of origin; this is known as the “healthy migrant effect” [14]. However, the health of immigrants tends to decline with time of residence in the new home country. The health among immigrants is complex, being influenced by the ethnic, cultural, and the economic diversity of the immigrants; the reason for migration; the migration process in itself; and the acculturation in the new home country [15].

### Theoretical framework

#### Health and social support

Self-care is demanding and may cause stress [16, 17]. High demands and low decision latitude are linked to stress and frustration that may cause impaired health [18]. Stress is thought to influence health, both by promoting behavioural coping responses that are detrimental to one’s health and by activating physiological systems [19]. However, levels of stress can be decreased or increased, depending on the kind of social support one is given and experiences [18]. Social support from a positive perspective can buffer stress and protect health.

Social support comprises structural and functional elements. The structural aspects of support refer to a social network, i.e., webs of social relationships and linkages that are best measured through quantitative scoring of the size of networks or existence of support resources (marital status, social networks, and community ties). The functional aspect is often differentiated in terms of three types of resources: instrumental, informational, and emotional [20]. *Instrumental* support involves the provision of material aid, for example, financial assistance or help with daily tasks. *Informational* support refers to the provision of relevant information intended to help the individual cope with current difficulties, typically taking the form of advice or guidance in dealing with one’s problems. *Emotional* support involves the expression of empathy, caring, reassurance, and trust, providing opportunities for emotional expression and venting [19].

### Previous studies concerning social support and diabetes mellitus

Social support provided by a family member or close friend has a buffering effect on distress; it is associated with greater diabetes management self-efficacy and better self-monitoring of blood glucose [1–4]. Conversely, conflicting perceptions about the ability to control diabetes and its chronic nature and congruous negative perceptions about diabetes symptoms among couples may sustain distress overtime [21]. Diabetes distress is defined as the worries, fears and threats (emotional distress) resulting from burdensome symptoms, onerous self-management regimens, fear of complications, and loss of functioning, and concerns about access to care [22]. It is an expected emotional response to having diabetes and is associated with poor diabetes self-management and glycaemic control, which also affects the family members.

The support from family members in relation to diabetes is associated with a range of factors such as e.g. race and ethnicity [6]; foreign-born PWDM, particularly those of non-European origin, are at risk of having lower knowledge about diabetes compared with native‐born persons [23]. Therefore, it has been emphasised that future research on social support for people living with diabetes should include ethnic groups [3]. The meaning of support, as described by foreign-born PWDM in Sweden, has been studied previously [16]. However, there is a need to explore the meaning of support as perceived, provided, and received by family-members, i.e. the individuals who are expected to provide support to foreign-born PWDM.

## Aim

This study explores the meaning of social support as described by close family members of foreign-born PWDM in Sweden. It also explores the support the family members provide, and the support they themselves need to be able to support the PWDM.

## Methods

### Design

The study had a qualitative explorative design and semi-structured interviews were performed for data collection. Semi-structured interviews may be favourable when studying a construct such as social support, as the method often takes into account gender and cultural variations whilst acknowledging there is something fundamental about social support regardless of these factors [4]. The method allowed the participants to guide the content within a framework of questions and aimed to reach a nuanced and deeper understanding of the meaning and implications of social support [24]. The focus of this study was to explore functional elements of social support [20]. The data collection also included quantitative demographic background data describing the study participants context. The Consolidated criteria for reporting qualitative research, the COREQ checklist [25], was used for study reporting.

### Local setting and procedure

In the area where the study was conducted, when patients are diagnosed with DM, they are referred to a hospital-based diabetes clinic for investigation and diabetes education. The subsequent care is delivered in primary healthcare centres, with referral to hospital‐based clinics for the management of any complications that are related to DM.

### Sample

A purposive sample of family members, as identified by foreign-born men and women, ≥ 18-years-old and diagnosed with type 2 diabetes, was recruited by the staff at a primary healthcare centre in an immigration-dense area in Sweden. To obtain a wide perspective of perceptions, we strove for variation in age, sex, and country of origin. Individuals who were interested in participating in the study completed a reply form that was forwarded to the researchers.

### Data collection

The data were collected between March 2021 and June 2023. The interviews were conducted by two female registered nurses (the authors) with several years of experience in caring for chronically ill patients, whereof one is also a diabetes specialist nurse (second author) with extensive experience in conducting research on migrants with diabetes mellitus. None of them were involved with the management of the clients or the healthcare centre. The researchers contacted presumptive respondents to introduce themselves, inform about the aim of the study, and arrange for the interviews. The need of interpreter was assessed and offered if a need was indicated on the reply form.

The plan was to conduct the interviews at the healthcare centre in a secluded room. However, due to the Covid-19 pandemic and the need for social distancing, the original plan was revised, and the interviews were held via telephone. The interviews lasted between 30 and 90 min and were audio-recorded. When needed (*n* = 3), an authorised interpreter of the same gender as the respondent was used and participated by telephone (three-partite conversation). Reflective notes were taken after each interview.

An interview guide was developed based on previous research within the area of social support [16, 17, 26]. Three open-ended questions focusing on the meaning of support, the support given by the relative to the PWDM, and the support needed by the relative to support the foreign-born PWDM formed the basis of the interview guide. Also questions on perceptions of diabetes mellitus (causes of diabetes and perspectives on future health and need of support), and about demographic data for contextualising information were included. The interview guide was pilot tested with two study persons (included in the study) and was found to function well. Throughout the interviews, probing questions, e.g. Can you tell me more? How did that make you feel? Could you give an example? were used to gain a deeper understanding of the participants’ perceptions and experiences [24].

### Data analysis

The text was analysed by using qualitative content analysis, which intends to describe and discover the variation in perceptions [27]. The interviews were transcribed verbatim by a professional secretary. Thereafter, to get an overview of the content, the authors read through the text several times. Each interview was considered as a unit of analysis, which was imported into NVivo (version 1.3 (535) QSR International Pty Ltd, 1999–2020) software. Text and phrases related to the study aim were identified; textual units were marked in the transcripts and thereafter coded, inductively. The codes were compared for similarities and differences between them. Groups of codes sharing mutual attributes were organised into sub-categories, which were then sorted into categories related to the theoretical framework i.e., functional elements of social support. Data that were not deductively covered by the theoretical framework [20] formed the basis for developing categories inductively. Hence, a mix of different basic procedures (inductive, deductive), as described by Mayring, was used [28]. In the final step of the analysis process, the findings were sorted under the themes in the interview guide. Data collection and analysis proceeded concurrently until no new data were added in the analysis. When comparing the statements from the different participants, it was based on openness for variation in the data and a search for patterns, regularities, and contradictions.

To increase the trustworthiness of the results, investigator triangulation, with an analysis of the data by two researchers independently, was used [24]. If needed, the results were discussed between the researchers until a consensus was reached. To reach confirmability, data are presented as categories with sub-categories, illustrated by illuminative quotations.

Descriptive statistics; numbers, median (range), were used for presenting demographic background data.

### Ethical considerations

The study was approved by the Swedish Ethical Review Authority (Dnr 2019–02555, 2020–05508). All participants provided recorded informed consent to participate, and the study was carried out in accordance with the Helsinki Declaration [29].

## Findings

### Characteristics of the study population

The study population included thirteen persons living in Sweden; seven were born in countries in the Middle East, one in Russia, and five in countries in Africa (Table 1). Their age range was 18–52 years (median 30 years). Most stated they were refugees, some had immigrated by family ties, and one due to work. Time of residence in Sweden was, on average, 9 years (range 1–30 years). All study participants, with two exceptions, were children of the PWDM, mainly daughters (*n* = 7), unmarried or divorced. Five of the respondents had no children or grandchildren of their own and lived together with the parent. The remaining two respondents were spouses of the PWDM. The number of children and grandchildren of the study participants varied (2–6 and 3–10, respectively). Most participants had secondary school and above educational level; three were unemployed and four were still students.

**Table 1:** Characteristics of the participants

Variable		*N* = 13
Age (years): median (range)		30 (18–52)
Gender (n)		
	Male	5
	Female	8
Country of birth (n)		
	Somalia	3
	Syria	2
	Palestine	2
	Lebanon	1
	Iraq	2
	Sudan	2
	Russia	1
Reason for migration (n)		
	Refugee	7
	Relative	5
	Labour	1
Time of residence in Sweden (years): median (range)		9 (1–30)
Family circumstances (n)		
	Unmarried/living alone	5
	Married/cohabitant	6
	Divorced	2
	Widowed	-
	Living with parents	5
Relationship with person with diabetes (n)		
	Spouse	2
	Child	11
Education (n)		
	Primary school ≤ 6 years	1
	Primary school ≤ 9 years	2
	Secondary school	6
	University > 2 years	4
Employment status: (n)		
	Gainfully employed	6
	Unemployed	3
	Student	4

Most of the PWDM had been diagnosed with diabetes abroad (*n* = 9), had the disease for an average of 10 years (range 1–46 years), and were treated with oral agents (*n* = 7). Some had complications related to diabetes, mainly eyes (*n* = 7) and foot/lower extremity (*n* = 5), but some also had issues with the heart (*n* = 4) and kidney (*n* = 2).

### Themes

The findings are structured around four main themes: (1) Perceptions of diabetes, (2) Meaning of Support, (3) Support provided to the PWDM, and (4) Need for support, with categories and sub-categories; see Table 2.

**Table 2:** Study themes with categories and sub-categories

Theme	Categories	Sub-categories
Perceptions of Diabetes Mellitus		
	Causes	
	Prognosis, the future	
Meaning of support		
	Social and emotional support	
	Perceived stressful situation	
	A chance to reciprocate or a filial piety	
Support provided to the PWDM		
	The level of support	
	Instrumental support	
		Administering medicines
		Economy and logistics
		Nutrition
		Basic care
	Informational support	
		Medical advice and support
		Navigating the healthcare system and translation
	Social and emotional support	
Need for support		
	Informative support Instrumental support Social network	

The family members described the meaning of support as being social and emotional. Several of them described their situation as stressful but considered caring for a family member with diabetes as a chance to reciprocate or a filial piety.

### Perceptions of Diabetes Mellitus

The family members perceived diabetes mellitus in the PWDM mainly caused by heredity and difficult life circumstances, but also lifestyle and lack of knowledge. Prognosis and future health were either seen as negatively influenced with increased demand of support due to development of complications or as unknown.

#### Causes

Regarding the cause of diabetes mellitus (DM), the majority discussed heredity, particularly as many had other family members or relatives with DM, but they also talked about the influence of difficult circumstances in life, such as war, migration, death, and sorrow. Also mentioned was concerns about relatives left behind as a potential trigger for the disease. Furthermore, lifestyle factors such as limited exercise and dietary habits with preferences for sweet things were viewed as causes for the disease. Finally, for some, the cause was unknown.

*… maybe genetic. There are others in the family who have had it. So my thoughts or the thoughts that I have is that it is genetic. (Respondent 2, R2)*

*…we’ve all moved… she was still there…and they don’t have a residence permit…thinking and thinking all the time…When there’s war in* [country of origin], *he got diabetes when he got scared. (R1)*

*I think mostly food and exercise. They rarely exercise…like sweet things…food. (R5)*

#### Prognosis, the future

Prognosis and the future were related to the perception that DM will influence health negatively and lead to complications, thus demanding more support. Others viewed the future as unknown, while a few expressed that lifestyle changes, increased exercise, and reduced intake of sugar might lead to better health.

*Well, of course I think the diabetes will affect her and give her other side effects or complications, I think. (R4)*

*I think they will need more support in the future, in a few years…in the future. Because it already feels like that, they are … tired. (R5)*

*It’s like other types of diseases, to avoid … sugar, and make sure you move a lot and … think about what you eat […] but I’m hopeful so probably better. (R9)*

### Meaning of support

The family members described the meaning of support as being social and emotional. Several of them described their situation as stressful but considered caring for a family member with diabetes as a chance to reciprocate or a filial piety.

#### Social and emotional support

Provision of social and emotional support, particularly when listening to the PWDM, meant both what was spoken and what could be perceived intuitively. Specifically, this related to talking about feelings and what had happened and also doing things together in the family to give a sense of meaning and prevent sadness.

.*feel from her voice, how she talks, if she feels well or not…important with language, to feel free…to be able to express yourself…and talk more about feelings, about things that happen…it’s a big help…so psychologically, just be…that we are there, can help. (R3)*

*be there and try to facilitate, support. We* (the family) *talk about everything…we tell him to be calm…help…take care of each other…. (R5)*

#### Perceived stressful situation

The majority of the study participants described their situation as difficult and burdensome; they experienced feelings of sadness and worry, often perceived as shared with the PWDM. For some study participants, knowing that the PWDM was not feeling well was stressful; for others, it felt like the PWDM had become dependent on them for help. The PWDM’s deteriorating health status led to increased demands in a situation where the family member had the responsibility for the PWDM in addition to already having a lot to deal with in their own lives. Furthermore, not having the choice to opt ​​out from being the care provider was perceived as stressful. The family members expressed that the PWDM was always on their mind and that they were always prepared for anything to happen. How they felt about the PWDM’s health status influenced them and their own health. They strove to decrease their need for support by giving the PWDM support for their well-being and health; sometimes, they sought help from their siblings who could relieve them.

*hard for me, hard, when they…when I see that they are not well…hard for me when they are not well…are having a hard time, in the hospital or mentally…I will be there, but I don’t know if I will be able to do it…when it only gets worse or it becomes more…I feel it is more demanding…It’s hard to find the time…hard that you already have a lot in your life…I can’t choose to let something go. (R5)*

*Worried that maybe her sugar level is changing…. You also think if you don’t hear anything from her, you think maybe something is happening to her…She doesn’t have such major illnesses and complications…But it’s in the mind…must feel for her voice, how she talks, if she’s well or not. (R3)*

*If she gets better, I get better too…He* (the brother) *who …lives in…*(overseas) *he can’t help her much. But my brother…works in a nursing home…he doesn’t have much time…But if she gets better, then it will be fine. If she gets better, then she won’t need as much help, or all day. (R1)*

Conversely, for some respondents, having a family member with diabetes was not perceived as stressful, because the family supported each other; others expressed it as “so it is” (R6), and becoming ill was viewed as natural.

#### A chance to reciprocate or a filial piety

Many of the family members expressed they did not need any support themselves and that supporting the PWDM was just part of their tradition, a filial piety to “help each other” and “take care of each other”. It was perceived as natural to help, e.g. one’s mother or husband, or seen as a chance to reciprocate, not the least when having knowledge, e.g. when being trained as healthcare staff. A few respondents stressed that it was the PWDM’s own disease, but their role as a family member was to provide support with basic information and, in particular, to remind him or her about appropriate food intake, medications, etc. In one case, the filial piety was described as having a negative influence as it had led to missed opportunities to accept job offers.

*It’s not a requirement…it’s my dad, help him so he doesn’t get other illnesses as a consequence. (R12)*

…*our tradition…when you have a sick man at home or a sick woman, we have to help each other. Take care of each other… (R6)*

*it’s my mother; she raised me, she gave birth to me and took care of me, and I won’t let anyone else take care of her. […] but I’ll be completely honest and say there are many internships and the like that I’ve had to turn down, precisely because I’ve had to help her and take care of her. (R4)*

### Support provided to the PWDM

The level of support provided by family members to the PWDM varied and was described both as instrumental or practical, informational and emotional, including social relations and networks.

#### The level of support

The level of support provided varied, but the majority described frequent contacts, often by telephone several times per day, also by grandchildren, or frequent visits. Some participants explained they were always with the PWDM doing things together, taking care of the person fully, whereas others considered the DM as their family member’s own disease and tried to see the person as normal and just supported him or her by reminding the PWDM to eat healthy.

.*almost here every day…I have my children here all the time…he makes sure to ask. (R12)*

*I usually contact dad every day, several times…I can also see my dad on the GPS…just in case something happens; if sugar is low or very high, then I know if he needs help so I can go to help him. (R 13)*

*…mostly with my mother…several times a day…I don’t have to talk to her for a whole hour. Sometimes we talk for an hour, sometimes we talk for five minutes. Sometimes I call her when I’m…on my way to work. (R1)*

*She lives together with us…I care for her and give her care… (R2)*.

#### Instrumental support

Instrumental support included a multitude of activities such as being involved in administering medicines, helping with the economy and logistics, nutrition, and providing basic care. Participants described actions such as giving reminders and providing support in *administering medicines*, both taking medications and ordering and picking up medicines from the pharmacy and contacting the healthcare centre for prescriptions as well as accompanying them to the visits to the physician.

*When a medication is finished, they tell me “now it is time to order” …Then I call the healthcare centre and make sure there is an interpreter…if I am free and not working I come along. (2)*

*take care of the medications …just give a reminder. (R7)*

Issues of *economy and logistics* were related to support given in paying bills and accompanying the PWDM, e.g. on the bus or giving him or her a ride to the healthcare centre, which was often a joint task for children in a family. Participants also discussed having contact with social services for the planning of home care.

*Even on the bus…help with tickets, with everything, with invoices…can you pass by…want to talk to you…can you order these* (medications), *also? Can you give me a ride there? Can you bring me there? (R1)*

*he* (the big brother) *takes mom to the hospital…or the emergency department when needed…I accompany her at the hospital. (R9)*

…contact with the home care. I had to talk to them…(R5).

Support regarding *nutrition* varied from reminders about the intake of an appropriate amount of food as well as the choice of food to others taking the full responsibility for planning and cooking all meals, as well as keeping an eye on the appropriate amount of food intake. An important issue raised was support regarding the choice of food and to answer the question: What is appropriate food?

*I take care of him. About his diet…remind him that some food isn’t good for him. (R7)*

*The diabetes* (blood sugar)*…goes down all the time and then goes up. It becomes a problem…problem knowing …”What should I take?”…We plan all the time that we will eat food that is not so much….sugar…vegetables are fine…we eat a little different now…my brothers, they want to lose weight…eating vegetables. I also want to lose weight. Mom doesn’t want to eat a lot of sugar. It’s like different, the whole family…everyone eats differently but I cook all the time…we have problems with the food…with what she can eat. (R9)*

In a few cases, the need for help with *basic care* and nursing, particularly foot care, was described.

*I take care of her… take her to the toilet. I help/support her with all her needs. I take her downtown. I manage her times. (R3).*

*to take care of her feet, and I put cream on them very often. She may sometimes get dry and a little numb, but no sores. (R4)*

#### Informational support

Informational support included medical advice and support and also navigating the healthcare system and translating. *Medical advice and support* mainly related to issues regarding blood glucose, such as instruction on how to use a glucometer or injection technique but also learning about reference values for normal blood glucose and managing hypoglycaemia. It also had to do with providing explanations, advice and answering questions, particularly among those trained in the healthcare professions.

*she asked me how to use this glucometer to be able to measure the blood glucose, so I read the instructions and helped her…She didn’t even know what the normal* (reference) *value was supposed to be. (R2)*

*He always did tests* (blood)*…he follows it…He asks* (daughter is a pharmacist) *“the test is like this; is that good? Is it ok?…” yes, but have you eaten…how much…And why is it like this. “Yes, grapes are not good…In this way, we talk all the time,…every day… (R12)*

*Navigating the healthcare system* was considered problematic for the PWDM, both due to language issues and difficulties understanding the healthcare system. The family members described how they supported the PWDM by accompanying him or her to the visits in healthcare, not the least to contribute with important *background* information to the healthcare provider, and to find/contact the right caregiver. They also helped to translate and answer telephone calls and messages from different caregivers.

*She needs help with everything…she also has questions…it has only been two years in here* (in Sweden)…*interpret when she goes to the doctor and the shop…on the bus…help with tickets…if she wants to go to the hospital…she cannot book it, she cannot find the way…unsure. She is scared… (R1)*

*they might not have an interpreter; they can demand our support. Me or my sisters help to interpret or we accompany her. She trusts more in our knowledge…from the 2nd or 3rd generation…we have been integrated; we have studied, understand the language, we have been educated…family members then will be a big support when it comes to information…language, culture,…healthcare staff will get a clearer picture of the patient’s background. (R3)*

Many of the participants described how they contributed with their own knowledge, either because they were trained in healthcare or what they had learnt from contacts with staff in healthcare. Thus, they described support in terms of acting as an extended arm of healthcare with what they knew or with knowledge they had acquired.

*I do have some knowledge. I also work at the hospital and have given…extra information…think about if I didn’t have the training or…didn’t have any knowledge about diabetes…there would be…a lack of care or support. (R3)*

#### Emotional and social support

To talk to, care for, be there and listen to the PWDM were considered important *emotional and social support*. The participants described planning and doing things together, often in the family, such as sharing meals, taking walks, and doing exercise, often to distract the PWDM’s from thoughts about their disease and their life situation, namely being an immigrant and living in a psychosocially burdensome life situation. Some talked about getting help at home from their daughters, living together, and not leaving the PWDM alone as important and promoting mental health.

*I listen to my mother… also help her with what she needs. She can also talk… what she is thinking and wondering about…she needs our help…my family. (R8)*

*We plan all the time that we will eat food that is not so much sugar…We walk together, do exercise at the same time…cook all the time… (R9)*

*She lives together with us…we don’t leave her…we support her…take her out …enjoying everyday life. I am always near her. (R4)*

### Need for support

The family members’ need for support was mainly focused on informative support, to increase knowledge about the disease, management, and self-care but also instrumental support with practical aspects in daily life. Information from professionals as well as peers was requested.

#### Informative support

The respondents expressed a clear need for more knowledge about the disease and self-care management, stating *‘to find out more’* (R1). The family members wanted to know all about the disease, particularly about the choice of food and what is considered appropriate, as well as blood glucose control, including reference values, measures in case of hypo-or hyperglycaemia, and self-monitoring. It was suggested that the information be given together with the PWDM, both from the time of diagnosis and onwards. The family members emphasised the importance of viewing the disease and the diseased person as a family matter and preventing the withholding of important information that was needed to be able to give appropriate support.

*find out about …the medication, …diet, how to exercise in the right way, exercise better…all about diabetes. (R2)*

*…about diabetes…I want to be informed, for example, about diet…As he* (the father) *does not know wat food is good for him; neither do I. Thus, I don’t know about the food, so I Google it always…alternative food…it would be very good to sit with a group of family members of others with diabetes. (R12)*

*it hurts relatives quite a lot not to know…siblings and mother, father they are quite important and close., very close people to us…strong relationship. When someone is not well and we don’t even know… You don’t need to know details, but to know how it will help the patient …(R3)*

Another important aspect raised was the wish to attend the PWDM’s visit with the physician and, thus, having the possibility to book appointments at suitable times themselves to be able to go with the PWDM to get information.

*She has the doctors…and asks…she meets the doctors…It would be good to know what is happening and …to get information. Yes, it’s because she knows how it goes… I can book the time for her* (at the healthcare centre*)…But often, the doctors send us a booked time. Interviewer: …So you would like to book it yourself, so you could join her? “Yes, yes, yes.” (R8)*

Some explained they sought information from other persons or family members having better knowledge, often due to their professional background (e.g. pharmacists, nurses, physicians, etc.). They also wished to arrange meetings with others in the same situation to exchange experiences and information in order to learn from each other. Others had tried to find information from the Internet, but there was a wish for both oral and written information materials.

*It happens that I call and ask a family member as I want them to ask, for example, a pharmacist…because with us it is different. We can go to the pharmacy and get advice as well. (R4)*

…*I want some more information regarding my father’s illness…you can search online. …how can I help him or what does the disease mean… what should we do to avoid more diseases… Maybe practical support too… regarding the food… Something in paper format… or… digital. Articles or something… (R13)*

#### Instrumental support

The need for instrumental support included support with practical aspects in daily life. Easy access to staff in diabetes care and healthcare in case of questions. They also raised the importance of having out of office hours and during weekends, suggesting open access through a dedicated telephone line.

*More access to care….if it’s the weekend, a later time. Because it’s only convenient times and weekdays…Perhaps there could be a line…now there is a line for certain diseases that you can call at any time, around the clock…the diabetes line. (R3).*

*It* (getting in touch with the health centre) *is very difficult. There is a phone queue….opens at eight; if I call at 10, then there are no available times, and you have to call another day….to get to X health centre, it is impossible to make an appointment with them… have to call…very problematic…(R13)*

Living together with the PWDM and not having to move between different places or to get peace and calm for the person were also described as important ways to offer support. Finally, the importance of having access to social care in the home, i.e. getting help with cooking and basic care needs, was emphasised.

#### Social network

Other family members, particularly children or siblings, and those being trained as healthcare staff were the main source of the social network for the study participants. They provided important emotional support by talking to each other. Other family members also provided instrumental support by helping out with different practical things. In a few cases, external support from work mates was described as a social network. Thus, in general, no other social support was desired.

*I have supported her…not so complicated, and I have family members that have better knowledge than me…My sister …she is studying to become a nurse…we are always here, the family… (R10)*

## Discussion

This study is unique as it explores the meaning of social support, support provided, and support needed, as described by close family members of foreign-born PWDM in Sweden. The main findings were that most study participants perceived the meaning of support as a chance to reciprocate or a filial piety, which implied frequent contacts: often daily (telephone or visits), in a family network, particularly children and siblings trained as health professionals. A situation characterised by feelings of sadness and worry (often mutual) for the PWDM’s ill-health and risk of developing complications added to a strained life situation from which one was not able to opt out. The support provided was mainly informational, but also instrumental/practical and emotional. Support needed by the family members had to do with information to increase knowledge about DM, particularly appropriate diet/food, and delivered together with the PWDM from the time of diagnosis and onwards.

### Support provided

This study clearly showed that a lot of support is provided by family members of foreign-born PWDMs. As previously stated, family members are a resource in health care and diabetes care, in particular [5], although they are underutilised, they are highly needed. The family members are mainly providing support with information, which they are requesting and seeking themselves. In the only previous study in this area [6], exploring the types of support accessed by Asians in the UK living with diabetes or coronary heart disease, the family was a source of emotional support and provided physical care; participants identified barriers and facilitators to the maintenance of a healthy lifestyle. In our study, there was a clear focus on appropriate diet/food and nutrition and how to support the PWDMs with this. A recurrent question was: What is appropriate food?

Instrumental support given varied from simple reminders on taking medications or intake of appropriate food to total care of all basic needs and the need to live together, demonstrating that Type 2 diabetes is a disease with many faces due to age and health status related to complications developed [16, 30]. This calls for attention to search for and assess individual beliefs and needs of support for family members, together with the PWDM. In person-centred care, as stated by law in Swedish health care [31, 32], family members also constitute an important collaborator, and represent the third party in the care triad [5].

Much of the support provided was related to the ability to communicate (e.g., procurement/administration of medications, paying bills, buying bus tickets, etc. via the internet, navigating/accessing healthcare, etc.). The ability to communicate and the need to use interpreters and have informational material in the native language have been found important for foreign-born PWDM, in relation to the need for support in DM, also affecting the entire life situation [16], which also need to be considered. Learning to manage diabetes is important from both the PWDM’s and the family members’ perspectives. However, to avoid misinterpretations due to limited knowledge or withholding of information to protect the PWDM it is important in all healthcare contacts to offer access to professional interpreters, instead of using family members [33]. Family members and friends are not trained as interpreters and are unlikely to have the appropriate medical vocabulary, leading to incomplete and inaccurate transmission of information. Incomplete information exchange during consultations, might affect clinical decision-making and can result in clinical errors; and are replete with clinical, social and ethical problems. Thus, they cannot be promoted as best practice.

### The meaning of support

In this study, similar to a previous study focusing on social support as described by foreign-born PWDM living in Sweden [16], the meaning of support was perceived medical and informational. PWDM expressed a clear information gap when diagnosed and limited information in general. In PWDM [16], in contrast to this study addressing family members, the focus on emotional support was limited. However, the study populations differ, as well as their background relating to migration (mainly labour migrants from the former Yugoslavia vs. refugees from the Middle East and Africa) and time of residence (Md 14.5 (3–50) vs. 9 (1–30) years) in the new host country. Furthermore, it is reasonable to assume that the needs and expectations from family members and PWDMs differ.

As previously described [34], several family members expressed giving support as being a chance to reciprocate or a filial piety. The respondents in this study originate from societies based on cultural values and norms where the focus is more on the group and where the family has an important role that might differ from a culture described as individualistic, as e.g. in Sweden [35]. In a situation where one is an immigrant, often having broken or limited social networks, the family support might be even stronger; it is, thus, important to consider and is a resource to be used in healthcare [5]. However, although the provision of support may be associated with a wish to comply with imported cultural norms and the care recipient’s wishes, it may also be associated with an awareness that inability or unwillingness to comply with the expectations of the family members and others would cause feelings of guilt and shame, often permanent [34]. This might leave the caregiver with little personal choice and control. Feelings of cultural obligation to provide care do not necessarily equate with willingness to care [36]. Limited or lack of knowledge of the disease and of available healthcare services, and preconceived ideas about the inappropriateness of support and public healthcare, may prevent informal caregivers from seeking or accepting formal support. This may contribute to the migrant caregivers’ vulnerability and needs to be considered [37]. Several respondents described a burdensome and stressful situation with frequent contacts, often daily by telephone or visits, characterised by feelings of sadness and concerns (often mutual) for the PWDM’s malaise and risk of developing complications, and frustration in trying to find appropriate information. In the demand-control-support model [18], this means a combination of high demands, low control, and limited support in caregiving, which are associated with high strain and risk of poorer physical and psychological health [38]. Imbalance between demands and a caregiver’s ability to cope with these contributes to high strain. Informal caregivers who report a heavy caregiver burden are at higher risk of negative health sequelae, such as depression [39, 40].

To cope with the situation and increase control, many used the family network, particularly children and siblings being trained in healthcare, to get informational support. It is illuminating to hear the descriptions on trying to access information e.g., by the Internet, but also the frustration of not finding appropriate material, either written or oral. As in previous studies of Middle Eastern migrants with diabetes or gestational diabetes, an information-seeking behaviour is mainly described [41, 42], in contrast to a more passive attitude of persons from Africa [23]. The difference might be that the studied population has a higher educational level, secondary school and above, compared to the mostly low educated in the previous study [23]. Thus, there is a good base and prerequisite for developing knowledge about diabetes mellitus, but support from healthcare staff with appropriate information is needed, particularly to buffer the stress perceived by the family members to prevent negative influence on health.

### Need for support

The focus on a need for information support indicates limited knowledge about DM and the body, as found in previous studies on foreign-born (migrants) compared to Swedish-born persons [23]. However, limited knowledge and a will to learn to manage the disease [16] are compensated by searching for material. This study shows that family members trained as healthcare staff and the Internet, are important sources of information. However, as previously shown [6], many are reluctant to using information from the internet both because it is not a trusted source of information, and because there is limited access to information in different languages. What is needed is support with access to valid and reliable information. In this study, the family members emphasised the importance of viewing the diabetes disease as a family matter. Thus, measures should be taken to provide adequate information about diabetes management and prevent the withholding of important information (without compromising the PWDM’s integrity) from the healthcare that is needed to be able to give appropriate support. This was particularly important at the time of diagnosis but also continuously, i.e., having the possibility to attend the visits with the physician in healthcare to get information. The family members wanted the opportunity to attend these visits together with the PWDM.

Respondents wished that learning about DM would take place both together with the PWDM, as well as with other family members and peers in the same situation, and by skilled staff in diabetes care. Group education, particularly in focus-group discussions based on individual beliefs and knowledge, can be a method to assess and meet individual needs in the person-centred care. This can be combined with exchanging information and letting the participants share their experiences of living with the chronic disease in order to learn to cope with it [43]. Feeling supported by peers in a safe environment is vital for PWDMs [44] and has the potential to promote health and prevent ill-health in a strained situation, also for family members. It is important to allow room for the family members to discuss their needs both individually and together with others in the same situation, without the PWDM being present. However, intervention studies delivering peer support by connecting informal care givers, such as family members to PWDM, are lacking [45]. Social support provided by a family member or close friend has a buffering effect on distress, and is associated with better diabetes management [1–4]. Nonetheless, conflicting perceptions about the ability to control diabetes and its chronic nature as well as congruous negative perceptions about diabetes symptoms among couples may sustain distress overtime [21] and thus, need to be acknowledged. It is important to consider that wrong or inadequate support from family members can affect the PWDM negatively. Also, there is a risk of negative influence on the family member who might be exposed to an unreasonably high burden because of the responsibility of being an informal caregiver.

The request to have a dedicated telephone line and open access to healthcare among the family members studied here should be met. Previous studies have shown that it can be difficult for migrants to navigate and to access healthcare, particularly due to language difficulties and a healthcare system based on telephone conversations, where patients must describe their symptoms to the healthcare provider before deciding on treatment options [46–48]. In fact, many adult migrants do not have appropriate health information and often face difficulties in managing health issues [49]. The health needs of migrants are a challenge for the healthcare system. Access to high-quality healthcare is particularly important for these individuals, as they face unequal medical treatment opportunities. All too often, migrants face serious inequalities in health status and inequality in access to healthcare and other important services [50].

### Limitations

Most of the informants were females (*n* = 8) and children of the PWDM, particularly daughters, which might be seen as a limitation. However, previously, it has been found that care and solitude delivered by next-of-kin in the home are mainly given by adult children, predominantly middle aged daughters [37]. In general, it is somewhat more common that women and persons born outside Europe are those caring for their next-of-kin in the home [51].

The study population mainly included individuals originating from the Middle East and Africa, being refugees and thus, represent the largest groups of non-European migrants in Sweden [13] also having the highest risk of developing type 2 diabetes [9, 10]. Most were educated above the primary school level, possibly related to being educated in Sweden. Hence, unanswered questions remain; specifically, what would the results have been in a population of recent European migrants and involving persons with lower education? The qualitative explorative approach, however, enabled a deeper understanding of the respondents’ experiences and beliefs and helped to disclose different perspectives. Thus, staff should meet patients in a person-centred manner [32], taking into consideration their individual beliefs, as well as the beliefs of their family members [5].

The data collection period was prolonged due to the Covid-19 pandemic, and the restrictions that followed led to the study being stopped for a period. The study design then had to be changed from face-to-face interviews to telephone interviews. It was further delayed because recruitment was done by staff in healthcare who were overloaded with work during the pandemic and thus, had limited time to help with the recruitment. However, telephone interviews worked well, even in cases when an interpreter was engaged. Informants appreciated having phone interviews, as they made it easy to attend and did not require transportation to the interview venue. Also, the topic for the interview was suitable for the method. A disadvantage was not being able to read the body language and emotional expressions during the interviews [24]. On the other hand, many informants really wanted to share a lot and expressed being grateful for being asked; the interviewers were left with positive impressions after the interviews, and the interview content was comprehensive.

The sample size might be seen as limited; however, redundancy level was reached in the data analysis and then a few extra interviews were conducted. Thus, sampling adequacy and data redundancy were achieved [24]. The most important limitation with the study design chosen is the inability to generalise from the results. However, the aim of the study was not to seek for explanations but to explore and to get a deeper understanding of the phenomenon studied. Since the data were carefully collected, analysed, and described, the results are transferable to groups and contexts with similar characteristics [24].

## Conclusions and implications

In conclusion, family-members of migrants with type 2 diabetes thought that supporting the PWDM was normal and a filial piety. Although it implied frequent contacts in a family network, with a particular emphasis on those trained as healthcare professionals, to get access to information, several family members expressed this was a chance to reciprocate. Support provided was not only informational, but it was also instrumental/practical and emotional in a life situation that was burdened with concerns for the PWDM developing complications. Support from the family members focused on nutrition and navigating the healthcare system. The family members emphasised the importance of viewing the disease and the PWDM as a family matter and wished health care staff not to withhold information from the family. There was a wish to accompany the PWDM to the physician visits to get information, requests for a booking system to make appointments at suitable times, and easy access to healthcare by having a dedicated telephone-line, and oral and written information, particularly on diet. Thus, there is a need to develop person-centred diabetes education, including the information material, to also include family members. It is also important to address structural issues with improved access to healthcare and information about the healthcare system. This would help family members with measures that increase their control. Hence, it would allow them to learn to live with and manage diabetes and prevent a negative trajectory in caregiving with perceived demands causing high levels of stress and strain.

## Data Availability

In order to protect the integrity, anonymity, and confidentiality of the respondents, data will not be shared, but are available from the corresponding author on reasonable request.
